# Supplementary material for: The Potential of Ultrasound Radiomics in Carpal Tunnel Syndrome Diagnosis: A Systematic Review and Meta-Analysis
Source: Diagnostics (Basel). 2023 Oct 23;13(20):3280. doi: 10.3390/diagnostics13203280 (PMC10606315; doi:10.3390/diagnostics13203280)
Supplement: Supplementary file 1 [file diagnostics-13-03280-s001.zip › diagnostics-2659607-supplementary.pdf]

Supplemental Material

Table S1. PRISMA Checklist

Section and Topic	#	Checklist item	Location
		**TITLE**	
Title	1	Identify the report as a systematic review.	Title
		**ABSTRACT**	
Abstract	2	See the PRISMA 2020 for Abstracts checklist.	Abstract
		**INTRODUCTION**	
Rationale	3	Describe the rationale for the review in the context of existing knowledge.	Introduction
Objectives	4	Provide an explicit statement of the objective(s) or question(s) the review addresses.	Introduction
		**METHODS**	
Eligibility criteria	5	Specify the inclusion and exclusion criteria for the review and how studies were grouped for the syntheses.	Methods
Information sources	6	Specify all databases, registers, websites, organisations, reference lists and other sources searched or consulted to identify studies. Specify the date when each source was last searched or consulted.	Methods
Search strategy	7	Present the full search strategies for all databases, registers and websites, including any filters and limits used.	Methods
Selection process	8	Specify the methods used to decide whether a study met the inclusion criteria of the review, including how many reviewers screened each record and each report retrieved, whether they worked independently, and if applicable, details of automation tools used in the process.	Methods
Data collection process	9	Specify the methods used to collect data from reports, including how many reviewers collected data from each report, whether they worked independently, any processes for obtaining or confirming data from study investigators, and if applicable, details of automation tools used in the process.	Methods

Data items	10a	List and define all outcomes for which data were sought. Specify whether all results that were compatible with each outcome domain in each study were sought (e.g., for all measures, time points, analyses), and if not, the methods used to decide which results to collect.	Methods
	10b	List and define all other variables for which data were sought (e.g., participant and intervention characteristics, funding sources). Describe any assumptions made about any missing or unclear information.	Methods
Study risk of bias assessment	11	Specify the methods used to assess risk of bias in the included studies, including details of the tool(s) used, how many reviewers assessed each study and whether they worked independently, and if applicable, details of automation tools used in the process.	Methods
Effect measures	12	Specify for each outcome the effect measure(s) (e.g., risk ratio, mean difference) used in the synthesis or presentation of results.	NA
Synthesis methods	13a	Describe the processes used to decide which studies were eligible for each synthesis (e.g., tabulating the study intervention characteristics and comparing against the planned groups for each synthesis (item #5)).	Methods
	13b	Describe any methods required to prepare the data for presentation or synthesis, such as handling of missing summary statistics, or data conversions.	Methods
	13c	Describe any methods used to tabulate or visually display results of individual studies and syntheses.	Methods
	13d	Describe any methods used to synthesize results and provide a rationale for the choice(s). If meta-analysis was performed, describe the model(s), method(s) to identify the presence and extent of statistical heterogeneity, and software package(s) used.	Methods
	13e	Describe any methods used to explore possible causes of heterogeneity among study results (e.g., subgroup analysis, meta-regression).	Methods
	13f	Describe any sensitivity analyses conducted to assess robustness of the synthesized results.	Methods
Reporting bias assessment	14	Describe any methods used to assess risk of bias due to missing results in a synthesis (arising from reporting biases).	Methods
Certainty assessment	15	Describe any methods used to assess certainty (or confidence) in the body of evidence for an outcome.	Methods
**RESULTS**			
Study selection	16a	Describe the results of the search and selection process, from the number of records identified in the search to the number of studies included in the review, ideally using a flow diagram.	Results<br>Figure 1

	16b	Cite studies that might appear to meet the inclusion criteria, but which were excluded, and explain why they were excluded.	Results<br>Figure 1<br>Table S3
Study characteristics	17	Cite each included study and present its characteristics.	Results,<br>Table 1<br>Table 2
Risk of bias	18	Present assessments of risk of bias for each included study.	Results
Results of individual studies	19	For all outcomes, present, for each study: (a) summary statistics for each group (where appropriate) and (b) an effect estimates and its precision (e.g., confidence/credible interval), ideally using structured tables or plots.	Results
Results of syntheses	20a	For each synthesis, briefly summarise the characteristics and risk of bias among contributing studies.	Results
	20b	Present results of all statistical syntheses conducted. If meta-analysis was done, present for each the summary estimate and its precision (e.g., confidence/credible interval) and measures of statistical heterogeneity. If comparing groups, describe the direction of the effect.	Results<br>Figure 3-5
	20c	Present results of all investigations of possible causes of heterogeneity among study results.	Results<br>Figure 5
	20d	Present results of all sensitivity analyses conducted to assess the robustness of the synthesized results.	Results<br>Figure 3,4
Reporting biases	21	Present assessments of risk of bias due to missing results (arising from reporting biases) for each synthesis assessed.	Results<br>Figure 5
Certainty of evidence	22	Present assessments of certainty (or confidence) in the body of evidence for each outcome assessed.	Results
**DISCUSSION**			
Discussion	23a	Provide a general interpretation of the results in the context of other evidence.	Discussion
	23b	Discuss any limitations of the evidence included in the review.	Discussion
	23c	Discuss any limitations of the review processes used.	Discussion

	23d	Discuss implications of the results for practice, policy, and future research.	Discussion
**OTHER INFORMATION**			
Registration and protocol	24a	Provide registration information for the review, including register name and registration number, or state that the review was not registered.	Methods
	24b	Indicate where the review protocol can be accessed, or state that a protocol was not prepared.	Methods
	24c	Describe and explain any amendments to information provided at registration or in the protocol.	Methods
Support	25	Describe sources of financial or non-financial support for the review, and the role of the funders or sponsors in the review.	Funding
Competing interests	26	Declare any competing interests of review authors.	Conflicts of interest
Availability of data, code and other materials	27	Report which of the following are publicly available and where they can be found: template data collection forms; data extracted from included studies; data used for all analyses; analytic code; any other materials used in the review.	Results

.

Table S2. Keywords and search results in different databases

Database	Keyword	Filter	Date	Results
PubMed	("radiomics" OR "radiomic" OR "ultrasound radiomics") AND ("carpal tunnel syndrome" OR "median nerve" OR "neuropathy" OR "nerve")	Title Abstract	September 17, 2023	34
Embase	("radiomics" OR "radiomic" OR "ultrasound radiomics") AND ("carpal tunnel syndrome" OR "median nerve" OR "neuropathy" OR "nerve")	Title Abstract	September 17, 2023	45
Web of Science	("radiomics" OR "radiomic" OR "ultrasound radiomics") AND ("carpal tunnel syndrome" OR "median nerve" OR "neuropathy" OR "nerve")	Abstract	September 17, 2023	30

Table S3. Excluded studies and reasons

Citations	Reasons
Rossi, F.; Bignotti, B.; Bianchi, L.; Picasso, R.; Martinoli, C.; Tagliafico, A.S. Radiomics of peripheral nerves MRI in mild carpal and cubital tunnel syndrome. La radiologia medica 2020, 125, 197-203, doi:10.1007/s11547-019-01110-z.	Focus on using magnetic resonance imaging for characterizing CTS
Wu, H.; Luo, B.; Zhao, Y.; Yuan, G.; Wang, Q.; Liu, P.; Zhai, L.; Lv, W.; Zhang, J. Radiomics analysis of the optic nerve for detecting dysthyroid optic neuropathy, based on water-fat imaging. Insights Imaging 2022, 13, 154, doi:10.1186/s13244-022-01292-7	Analyze the optic nerve
Zha, J.; Ni, J.; Chen, S.; Feng, H.; Che, T.; Qiao, S. Ultrasound Radiomics-Guided Iliac Fascia Block on Postoperative Cognitive Dysfunction in Elderly Patients Undergoing Hip Surgery. Comput Math Methods Med 2022, 2022, 2492667, doi:10.1155/2022/2492667.	Target on nerve block
Hashiba, J.; Yokota, H.; Abe, K.; Sekiguchi, Y.; Ikeda, S.; Sugiyama, A.; Kuwabara, S.; Uno, T. Ultrasound-based radiomic analysis of the peripheral nerves for differentiation between CIDP and POEMS syndrome. Acta Radiol 2023, 2841851231181680, doi:10.1177/02841851231181680.	Emphasize differentiating demyelinating peripheral nerve neuropathy
Wang, M.Y.; Jia, C.G.; Xu, H.Q.; Xu, C.S.; Li, X.; Wei, W.; Chen, J.C. Development and Validation of a Deep Learning Predictive Model Combining Clinical and Radiomic Features for Short-Term Postoperative Facial Nerve Function in Acoustic Neuroma Patients. Curr Med Sci 2023, 43, 336-343, doi:10.1007/s11596-023-2713-x.	Predict facial nerve function in acoustic neuroma patients
